# Europäische Dateninfrastrukturen: Ansätze und Werkzeuge zur Nutzung von Daten zum Wohl von Individuum und Gemeinschaft

**DOI:** 10.1007/s00287-021-01386-4

**Published:** 2021-08-05

**Authors:** Boris Otto, Anja Burmann

**Affiliations:** 1 Fraunhofer-Institut für Software- und Systemtechnik ISST, Emil-Figge-Straße 91, 44227 Dortmund, Deutschland; 2 Lehrstuhl für Industrielles Informationsmanagement, Technische Universität Dortmund, Joseph-von-Fraunhofer-Str. 2–4, 44227 Dortmund, Deutschland

## Abstract

Daten stellen eine strategische Ressource für die Wettbewerbsfähigkeit von Unternehmen und die Prosperität der Gesellschaft dar. Von der Nutzung von Daten vieler einzelner Akteure profitieren die Gemeinschaft, aber auch das Individuum. Beispiele hierfür liefern das Gesundheitswesen oder die Mobilität. Dabei sind die Interessen der Individuen in Bezug auf Datenschutz und Datensouveränität über Nutzungsvereinbarungen hinaus zu wahren und bestenfalls technologisch sicherzustellen. Datenräume, basierend auf verteilten Dateninfrastrukturen, stellen Datendienste und Datennutzungsregeln für Individuen und Organisationen bereit. Beispiele hierfür liefern die International-Data-Spaces(IDS)-Initiative oder die Initiative Gaia‑X zur Schaffung einer verteilten Dateninfrastruktur in Europa. Instanziierungen in den Bereichen Mobilität oder Smarthome zeigen Vorteile von Datenökosystemen für Individuen, die Gemeinschaft und die Gesamtheit der Dienstanbieter. Gleichzeitig werden Risiken wie die geeignete Interessensvertretung einzelner Nutzender deutlich. Anhand zweier Fallstudien werden offene ethische, rechtliche, betriebswirtschaftliche und technische Fragen bei der verteilten Umsetzung von Datenräumen und Dateninfrastrukturen unter Sicherstellung europäischer Wertevereinbarungen aufgezeigt.

## Datennutzung und Datensouveränität

Dass Daten eine strategische Ressource für Wirtschaft und Gesellschaft darstellen, ist Konsens zwischen allen Interessensgruppen. So profitiert die Allgemeinheit in vielen Fällen davon, wenn vorhandene Daten besser genutzt werden. Zahlreiche Beispiele finden sich im Gesundheitswesen, wenn Daten einzelner Patienten aggregiert und ausgewertet werden können, um wirksamere Medikamente zu entwickeln, und im Verkehrswesen, wenn Daten einzelner Reisender zur verbesserten Lenkung von Verkehrsströmen sowie für integrierte, intermodale Mobilitätsdienste verwendet werden können. Beide Beispiele zeigen, dass auf Basis von Daten Leistungsangebote durch Unternehmen individualisiert werden, aber auch öffentliche Dienste bedarfsgerechter geplant und bereitgestellt werden können. Innovation aus Daten entsteht zumeist, wenn Daten verschiedener Datenquellen und Kontextdaten kombiniert und analysiert werden. Ein intermodaler Mobilitätsdienst erfordert z. B. Fahrplandaten verschiedener Verkehrsträger, Bewegungsdaten vieler Reisender sowie Angaben zu Staus, zu Störungen auf Bahnstrecken oder zu Großveranstaltungen. Die Daten vieler einzelner Akteure bieten also großes Potenzial für Mehrwert für die Allgemeinheit und durch einen verbesserten Service auch für das Individuum. Gleichzeitig sind aber die Interessen des Einzelnen am Datenaustausch, der Nutzung der Daten durch Dritte und in Bezug auf Anonymität der eigenen Person zu wahren [12].

Die gesellschaftliche Diskussion rund um die Nutzung von Daten ist vielfach dominiert von Extrempositionen. Personenbezogene Daten unterliegen strengen Datenschutzauflagen, was die Nutzung individueller Daten selbst in Fällen offenkundigen öffentlichen Interesses der Gemeinschaft wie auch des Individuums, wie der Bewältigung der aktuellen SARS-CoV-2-Pandemie, verhindert. Zugleich sind Individuen jedoch an der Privatheit ihrer Daten paradoxerweise kaum interessiert, wenn sie soziale Netzwerke nutzen [14]. In Bezug auf nichtpersonenbezogene Daten erregte das sogenannte „Daten-für-Alle-Gesetz“ hohe Aufmerksamkeit bei Unternehmen. Es fordert u. a. die Nutzung von nichtpersönlichen Daten als Gemeingut sowie eine Datenteilungspflicht für marktdominierende Unternehmen [18].

Der Schutz des Individuums auch in Bezug auf Daten wird in der Europäischen Union hoch priorisiert, was die Europäische Datenschutzgrundverordnung (EU-DSGVO) zeigt. Die zentrale Aggregation von personenbezogenen Daten analog zu Vorbildern außereuropäischer Wirtschaftsräume soll erschwert bzw. ganz unterbunden werden. In diesem Kontext sind Datenräume und Dateninfrastrukturen softwaretechnische Konzepte für den Ausgleich individueller und gemeinschaftlicher Interessen. Sie zielen auf Werkzeuge ab, die Daten für das Gemeinwohl und den Vorteil des individuellen Datengebenden nutzbar zu machen, und dabei die Interessen der Datenbereitstellenden zu wahren, ohne deren Datensouveränität unverhältnismäßig einzuschränken. Sowohl die europäische Datenstrategie [7] als auch die Datenstrategie der Bundesregierung [3] fordern daher den Aufbau von Datenräumen auf Basis einer verteilten Dateninfrastruktur. Das strategische Ziel ist in beiden Fällen der Ausgleich gemeinsamer gesellschaftlicher und wirtschaftlicher einerseits und individueller, privater Interessen andererseits. Datengebende sollen in die Lage versetzt werden, unter fairen Bedingungen an der Datenökonomie teilhaben zu können, indem sie Nutzungsbedingungen für die Daten artikulieren, die Datennutzung nachvollziehen und Nutzungsbedingungen in Grenzen auch technisch durchsetzen können.

Der Begriff der Datensouveränität erfährt zurzeit viel Aufmerksamkeit, und es hat sich eine Vielzahl unterschiedlicher Interpretationen herausgebildet (etwa als Gesetz oder als Recht) [12]. In Verbindung mit Datenräumen und Dateninfrastrukturen bezeichnet Datensouveränität die Fähigkeit einer juristischen oder natürlichen Person zur Selbstbestimmung über ihre Datengüter [21]. Denn Datenräume und Dateninfrastrukturen zielen darauf, die Fähigkeit der Datensouveränität softwaretechnisch zu unterstützen.

Die Gestaltung dezentraler, föderierter Dateninfrastrukturen durch ein Zusammenspiel aus Wirtschaft und Gesellschaft ist nicht trivial, und bedarf vorreitender Initiativen, anhand derer Erkenntnisse und Gestaltungsprinzipien sich andere Anwendungsbereiche auf den Weg machen können. Anhand zweier Fallstudien, dem Datenraum Mobilität und der Digital Life Journey, wird die Anteilnahme und Notwendigkeit zur Integration von Wirtschaft, öffentlicher Hand und Individuen in die Gestaltung von Datenräumen aufgezeigt. Beide vorgestellten Ansätze sind exemplarischer Natur, im Falle des Datenraums Mobilität durch die Bundesregierung und im Falle der Digital Life Journey durch die Fraunhofer Gesellschaft initiiert.

Dieser Beitrag verfolgt das Ziel, technologische Ansätze für die Sicherstellung europäischer Werte wie Datenschutz und Datensouveränität beim Aufbau von Datenräumen aufzuzeigen. Daraus sollen Handlungs- und Forschungsbedarfe beim Aufbau und bei der Nutzung von Dateninfrastrukturen auf Basis von Datenräumen identifiziert werden. Dazu legt das nächste Kapitel die Grundlagen und beschreibt den Stand der Technik, bevor im Anschluss daran 2 Fallstudien aktuelle Praxiserfahrungen schildern. Die Analyse der Fallstudien erlaubt dann die Identifikation von Handlungs- und Forschungsbedarfen.

## Stand der Technik

### Datenräume und Dateninfrastrukturen

Ein Datenraum ist ein Datenintegrationskonzept, das im Wesentlichen durch 4 Merkmale gekennzeichnet ist [9, 11].

- Erstens erfordert ein Datenraum keine physische Integration der Daten, sondern die Daten werden verteilt gehalten. Ein Datenraum basiert also auf einer verteilten Datenhaltungsarchitektur.
- Zweitens sieht ein Datenraum kein gemeinsames Datenbankschema vor, sondern die Integration der Daten erfolgt auf semantischer Ebene, vorzugsweise unter Nutzung von Vokabularen.
- Drittens unterstützt ein Datenraum die Vernetzung von Daten auf Basis von Linked-Data-Konzepten, wo verteilte Daten per Uniform Resource Identifier (URI) kodiert eindeutig identifiziert und miteinander verknüpft sind. Dies hat auch redundante Datenhaltung zur Folge.
- Schließlich können Datenräume viertens ineinander verschachtelt und überlappend sein. Daraus folgt auch, dass Datenraumteilnehmende ihre Daten in verschiedenen Datenräumen verfügbar machen können und dass Daten zwischen Datenräumen geteilt werden können.

Eine Dateninfrastruktur stellt für einen bestimmten Gesellschaftsbereich wie die Mobilität oder für eine gesamte Volkswirtschaft Daten, Datendienste und Regeln für die Datennutzung für Individuen und Organisationen bereit [6]. Durch die Nutzung der Dateninfrastruktur durch Datengebende, Datennutzende sowie Intermediäre entstehen Datenräume. Dateninfrastrukturen bilden die informationstechnische Basis für Datenräume.

Aus den verschiedenen Interaktions- und Austauschbeziehungen der Datenraumteilnehmenden entstehen Datenökosysteme. Sie ermöglichen die Wiederverwendung der Daten, die Integration von Datennutzenden und Datengebenden und damit die Verknüpfung von Daten zu innovativen Diensten [10].

**Figure 1:**
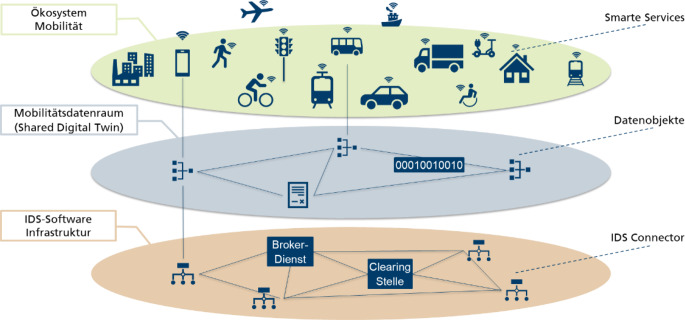

Abb. 1 zeigt den Zusammenhang zwischen Dateninfrastrukturen, Datenräumen und Datenökosystemen am Beispiel der Mobilität.

### Softwarearchitekturen für Datenräume

Das Konzept der Datenräume wird umgesetzt in Datenplattformen mit verteilter Datenhaltung und bilateralem Datenaustausch.

Die International-Data-Spaces(IDS)-Initiative^1^ zielt auf die Steigerung von Interoperabilität und Datensouveränität beim Teilen von Daten sowie auf den Vertrauensschutz von Datengebenden und Datennutzenden ab. Die Initiative startete 2015 mit einem durch das Bundesministerium für Forschung und Bildung (BMBF) geförderten Projekt der Fraunhofer-Gesellschaft, gefolgt Anfang 2016 durch die Gründung eines gemeinnützigen Vereins (International Data Spaces Association), in dem sich mittlerweile über 120 Unternehmen (Anwender und Technologieanbieter) aus mehr als 15 Ländern organisieren.

Die International Data Spaces Association spezifiziert im IDS-Referenzarchitekturmodell (IDS-RAM) [20] eine verteilte Softwarearchitektur für das Teilen und den Austausch von Daten zwischen Teilnehmenden eines Datenraums.^2^

**Figure 2:**
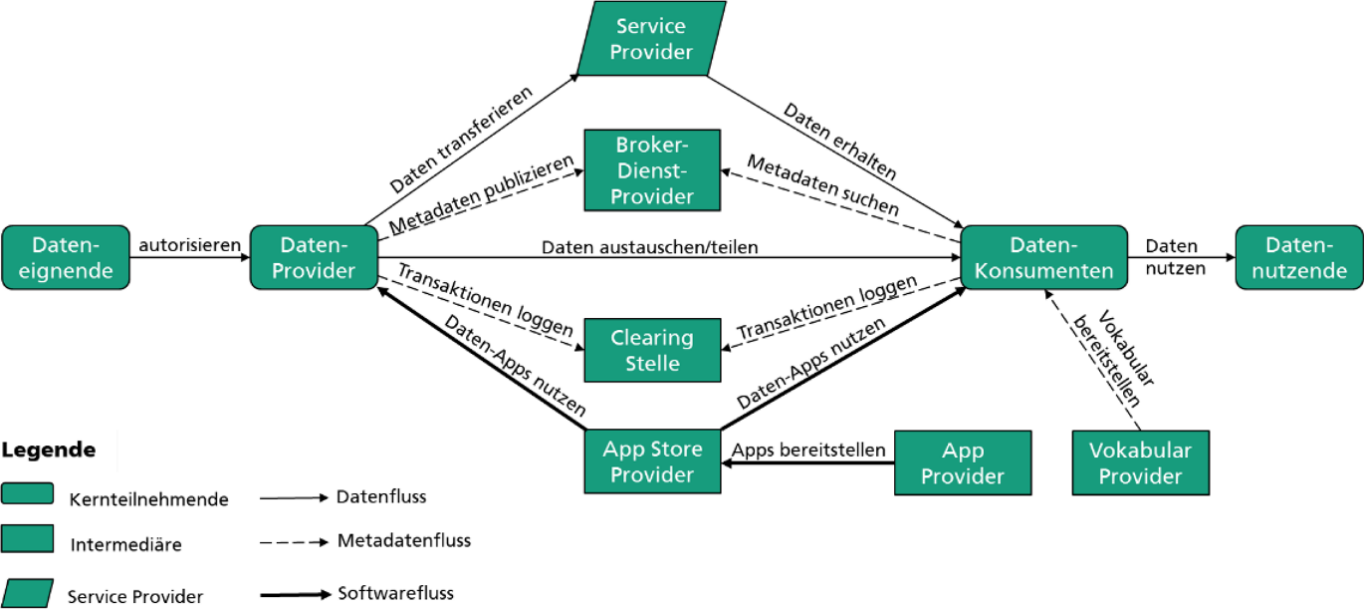

Das zugehörige Rollenmodell (siehe Abb. 2) unterscheidet zwischen Kernteilnehmenden, also Datenbesitzenden und Datengebenden einerseits und Datenempfangenden bzw. Datennutzenden andererseits. Der Datenaustausch findet nur zwischen Datengebenden und Datenempfangenden statt. Eine zentrale Datenhaltung ist nicht vorgesehen, und es hat auch keine zentrale Stelle Zugang zu den ausgetauschten Daten, was Vorteile z. B. in Bezug auf Datenschutz und Datensouveränität (sowohl von Individuen als auch von Organisationen), aber auch Verfügbarkeit und Effizienz bietet. Der bilaterale Datenaustausch umfasst die Wirkdaten einerseits und Metadaten andererseits. Erstere stellen die Daten selbst dar, in Anwendungsfällen im Gesundheitswesens z. B. persönliche Daten wie Geschlecht, Geburtsdatum, eingenommene Medikamente oder die Krankheitshistorie. Metadaten beschreiben diese Daten in eben den genannten Kategorien und spezifizieren die Nutzungsbedingungen, die Datengebende für die Verwendung ihrer Daten durch Datenempfangende vorgeben.

Die Vermittlung zwischen Datengebenden und Datenempfangenden übernimmt ein Broker-Dienst, dessen zentrale Aufgabe die Verbindung zwischen Datenangebot und Datennachfrage ist. Datengebende beschreiben im Broker ihre Daten über Metadaten, die im Informationsmodell des IDS-RAM definiert sind. Datenempfangende können den entstehenden Datenkatalog durchsuchen und bei Bedarf Daten direkt vom Datengebenden anfragen. Das Informationsmodell des Brokers folgt der W3C-Empfehlung „Data Catalog Vocabulary“ (DCAT) [25].

Eine Clearing-Stelle überwacht die Datentransaktionen, sodass die beteiligten Parteien Transparenz darüber haben, ob die Datentransaktion erfolgreich war, ob also sowohl Wirkdaten als auch Nutzungsbedingungen korrekt ausgetauscht wurden.

Ein App Store bietet die Möglichkeit, standardisierte Datendienste (z. B. Transformationsdienste zur Überführung von Daten aus einem Quell- in ein Zielschema sowie Datenanalysedienste) im Datenraum verfügbar zu machen. Die Datendienste werden als isolierte Anwendungscontainer bereitgestellt, z. B. über die Docker-Schnittstelle.

Ein gemeinsames Vokabular zur Beschreibung von Ressourcen auf Basis des ursprünglichen Standards zur Beschreibung von (Meta‑)Daten, dem Resource Description Framework (RDF), bildet ein gemeinsames semantisches Informationsmodell im Datenraum. Dieses kann von den Datenraumteilnehmenden gemeinsam gepflegt werden. Da semantische Interoperabilität erst möglich ist, wenn die datenerzeugenden und datenverwendenden Dienste bekannt sind, beinhaltet das IDS-Informationsmodell sowohl die Daten selbst als auch Datendienste zur Bereitstellung bzw. zum Zugang der Daten [23].

Die wichtigste Softwarekomponente des IDS-RAM ist der IDS Connector.^3^ Der IDS Connector stellt die Verbindung zu den Datenquellen der Datengebenden her, verwaltet Metadaten zu den Datenquellen und zu den Nutzungsbedingungen der Daten und sendet bzw. empfängt die Daten inkl. der Nutzungsbedingungen.

Darüber hinaus ist er in der Lage, Nutzungsbedingungen zu interpretieren und technisch durchzusetzen. Die Durchsetzbarkeit der Nutzungsbedingungen wird erreicht, indem der IDS Connector vor unerlaubtem Zugriff durch Dritte geschützt ist, indem Anwendungen durch Containervirtualisierung isoliert werden und Nutzungsbedingungen maschinenlesbar sind.

**Figure 3:**
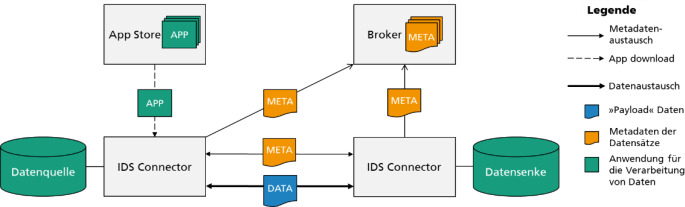

Schließlich stellt der IDS-Connector beschreibende Daten für den Broker-Dienst bereit. Abb. 3 zeigt die Kernaufgaben des IDS Connectors in seinem Zusammenspiel mit dem App-Store- und Broker-Dienst.

Die Nutzungsbedingungen bilden sozusagen die „AGB der Datenökonomie“, da sie regeln, unter welchen Bedingungen geteilte Daten vom wem genutzt werden dürfen. Die IDS-Initiative hat im ersten Schritt 14 Regelklassen definiert [5]. Beispiele sind:

- Datennutzung erlauben/verbieten
- Datennutzung beschränken auf Nutzergruppen bzw. Nutzungszwecke
- Datennutzung bei Eintritt eines bestimmten Ereignisses beschränken
- Datennutzung innerhalb eines bestimmten Zeitintervalls erlauben
- Datennutzung *n*-mal erlauben
- Daten nach Nutzung löschen
- Datennutzung protokollieren

Die Nutzungsbedingungen basieren auf der Open Digital Rights Language (ODRL) und ermöglichen so eine interoperable Beschreibung, Interpretation sowie Durchsetzung der Regeln.

Ebenso wenig wie es hundertprozentige Datensicherheit gibt, ermöglicht der IDS Connector keine hundertprozentige Datensouveränität. Beispielsweise sind Nachverfolgbarkeit von Datentransaktionen und Durchsetzbarkeit von Nutzungsbedingungen nur in Anwendungen möglich, die innerhalb des IDS Connectors ausgeführt werden. Wenn die Daten eine Systemkette von IDS Connector-Implementierungen verlassen, kann beides nicht gewährleistet werden. Ziel ist vielmehr, die Grenze dessen, was technisch zur Wahrung der Datensouveränität möglich ist, zu verschieben, sodass sich Datengebende nicht allein auf vertragliche und gesetzliche Regelungen verlassen müssen.

Während die IDS-Initiative auf Datensouveränität i. e. S. abzielt, also das Teilen und die gemeinsame Nutzung der Daten fokussiert, ist Datensouveränität i. w. S. Gegenstand der Initiative GAIA‑X^4^ zur Schaffung einer verteilten Dateninfrastruktur in Europa. Zusätzlich zum IDS-Fokus umfasst GAIA‑X auch die Souveränität über Daten bei der Speicherung der Daten und in Bezug auf die Cloud-Infrastruktur, auf der die IDS-Komponenten ausgeführt werden.

GAIA‑X wurde von der deutschen und der französischen Regierung initiiert. Die kürzlich gegründete gemeinnützige Einrichtung mit Sitz in Brüssel hat Mitgliedschaftsanträge von mehr als 200 Organisationen aus der ganzen Welt. Sie zielt auf die Spezifikation der GAIA-X-Gesamtarchitektur ab, auf den Aufbau einer Open Source Software Community für die Kernkomponenten der Infrastruktur sowie auf die Bereitstellung von Test- und Zertifizierungsverfahren. Die Komponenten des IDS-RAM werden derzeit in die GAIA-X-Architektur integriert [13].

### Personal-Information-Management-Systeme

Die Erschließung persönlicher Daten zum Wohle der Gemeinschaft und gleichzeitig die Sicherstellung von Nutzen für Datengebende führt über die Einbindung des Individuums. Die Verwaltung und Organisation persönlicher Daten sowohl für berufliche als auch private Zwecke ist seit einigen Jahren Gegenstand der Forschung [24]. Beispiele persönlicher Daten sind Identitätsangaben, Fotos, Kalendereinträge sowie Einträge auf Social-Media-Plattformen. Personal-Information-Management(PIM)-Systeme sind in diesem Zusammenhang Methoden und Prozeduren, um Informationen auf einer alltäglichen Basis zu verarbeiten, zu kategorisieren und abzurufen [15]. Im Zuge der fortschreitenden Digitalisierung des Alltags und des Aufbaus von Datenökosystemen präzisieren Abitedoul et al. ein PIM-System als eine Datenbank, um alle entlang des Lebens um ein Individuum herum entstehenden digital verfügbaren Informationen zu halten und einen Zugriff darauf zu organisieren [1].

Aktuelle Herausforderungen beim Management persönlicher Daten sind mangelnde Transparenz über die Speicherung und Verwendung persönlicher Daten durch Dritte, mangelnde Kontrolle über die Datennutzung sowie Lock-in-Effekte auf digitalen Plattformen. Viele Web-Services-Anbieter nutzen proprietäre Protokolle und Schnittstellen (engl. Application programming interfaces, APIs) für den Datenzugriff, sodass Nutzende ihre Daten nicht einfach serviceübergreifend integrieren und portieren und Entwickelnde keine plattformübergreifenden Anwendungen bereitstellen können.

Die Datenschutzgrundverordnung der Europäischen Union (EU-DSGVO) stärkt zwar die Position Einzelner in Bezug auf persönliche und personenbezogene Daten, aber dennoch wächst die Dominanz von Plattformanbietern, die eine Vielzahl persönlicher Daten aggregieren und analysieren, um Dienste anzubieten, ohne dass einzelne Datengebende faktisch eine Möglichkeit haben, dies zu verhindern und trotzdem das Dienstangebot zu nutzen. Daneben sind wirtschaftliche Weiterverwendung durch Dienstanbietende selbst oder Dritte und potenziell missbräuchliche Nutzung von Daten für Individuen kaum nachvollziehbar. Daher werden Forderungen laut nach Datentreuhändern, „Data Trusts“, um die Interessen der Vielzahl der einzelnen Nutzenden zu bündeln und ein wirksames Gegengewicht zu den Plattformanbietern zu bilden [22].

Lock-in-Effekte zu vermeiden und dem Individuum die Kontrolle über die eigenen Daten zurückzugeben, sind Ziele des Projekts SOLID (abgeleitet von Social Linked Data),^5^ was die Hoheit von Nutzenden über die eigenen Daten in den Fokus stellt und von den Initiatoren des World Wide Web selbst als Antwort auf zunehmend destruktive Nutzung ebendessen gegründet wurde. Der Vision einer „menschzentrierten Technologie“ folgend, schafft SOLID Konventionen und Werkzeuge, um dezentrale, modulare und erweiterbare Anwendungen zu entwickeln. Kern sind „Personal Online Data Stores“, sogenannten Pods, die beliebig gefüllt und gehostet werden können. Über ein Authentifizierungs- und Berechtigungskonzept können Daten angefragt und zur Nutzung freigegeben werden. Nutzende behalten dabei sowohl die Hoheit als auch Kontrolle über ihre Daten [16].

Attoresi und Moraes geben einen aktuellen Überblick zu PIM-Systemen sowie aktuellen Herausforderungen und Entwicklungen [2].

## Fallstudien

### Datenraum Mobilität

Die Initiative zum Datenraum Mobilität in Deutschland wurde vom Bundeskanzleramt initiiert und zielt auf einen vertrauenswürdigen Datenraum ab, in dem verschiedene Teilnehmende Daten teilen, um gemeinsam innovative Mobilitätsdienste zu entwickeln. Angestrebt ist ein innovatives Datenökosystem wie in Abb. 1 dargestellt.

Der Datenraum Mobilität bildet eine verteilte Plattform zum Austausch, Teilen und zur gemeinsamen Nutzung von Daten und schafft somit einen mehrseitigen Markt für Daten. Marktseiten sind Datengebende, Datennutzende sowie Anbietende von intermediären Diensten. Beispiele sind:

- Mobile Bürger
- Start-up- und kleine Unternehmen als Anbieter von intermodalen Mobilitätsdiensten (z. B. door2door, FREE NOW)
- Öffentliche Nahverkehrsbetriebe
- Eisenbahnverkehrsunternehmen und Luftfahrtunternehmen
- Automobilhersteller als Datengebende für „Connected-Car“-Daten
- Datenmarktplätze wie die MDM-Plattform der Bundesanstalt für Straßenwesen (BASt)
- Datenaggregatoren und -konzentratoren wie HERE
- Öffentliche Datenquellen wie der Deutsche Wetterdienst (DWD)

**Figure 4:**
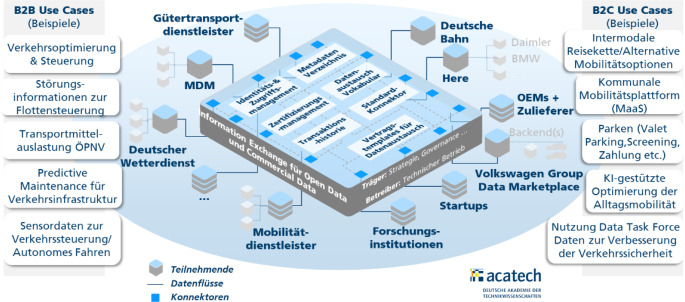

Abb. 4 zeigt die Gesamtarchitektur des Datenraums Mobilität mit den verschiedenen Datenraumteilnehmenden.

Informationstechnischen Kern bildet eine verteilte Softwareplattform auf Basis des IDS-RAM. Die Plattform umfasst ein Identitäts- und Zugriffsmanagement, ein Metadatenverzeichnis, das Zertifizierungsmanagement, ein Datenaustauschvokabular, eine Transaktionshistorie, ein Standard-Connector-Angebot sowie Vorlagen für Datenaustauschverträge. Die Plattform befindet sich zurzeit im Aufbau. Es werden parallel Anwendungsprojekte umgesetzt, und die Plattform wird im Laufe des Jahres 2021 an einen Betreiber übergeben.

Beispiele für Anwendungsprojekte sind:

- Durchgängige intermodale Navigation und Buchung für mobile Bürger vom Start bis zum Zielpunkt der Reise
- Individualisierte Versicherungsangebote für die Mobilität
- Innovatives Parkmanagement
- Steuerung von Verkehrsströmen sowohl auf kommunaler als auch überregionaler Ebene

Als Trägerorganisation ist eine nichtgewinnorientierte Unternehmung geplant, die offen ist für Beteiligung aller Interessensgruppen. Das steht im Einklang mit dem Ziel der Initiative, für dividuelle Geschäftsmodelle von Dienstanbietenden eine Infrastruktur zur Verfügung zu stellen – ähnlich wie das Autobahnnetz eine Infrastruktur für die Geschäftsmodelle von Fernbusunternehmen und Logistikdienstleistern bildet.

### Digital Life Journey

Die Gesamtheit der von Individuen hinterlassenen digitalen Daten bildet ein digitales Abbild des Individuums. Dieses digitale Abbild durchläuft verschiedene Entwicklungsstufen, deren Gesamtheit die „Digital Life Journey“^6^ bildet [17].

- Der „Digitale Schatten“ beschreibt die digitalen Spuren, die aus Sicht des Individuums häufig unbewusst, ungesteuert und nicht integriert in der digitalen Umwelt hinterlassen werden.
- Das „Digitale Ich“ kennt Art und Umfang der digitalen Spuren innerhalb eines Datenökosystems und stellt Technologien und Werkzeuge bereit, Datenverwendungen unter Erhöhung der Datensouveränität zu steuern.
- Der „Digitale Zwilling“ löst sich zusätzlich von seinem „Erzeuger“ und erlernt Fähigkeiten, um in enger Abstimmung mit seinem realweltlichen Zwilling autonom mit dem Datenökosystem zu interagieren.

Die Digital Life Journey zielt darauf ab, das digitale Abbild in seiner Gesamtheit entlang seiner zeitlichen Existenz und Entwicklungsstufen zu beschreiben und dabei technologische, gesellschaftliche, rechtliche, ökonomische und ethische Aspekte zu berücksichtigen. Die Digital Life Journey liefert Konzepte und Werkzeuge für die Datensouveränität Einzelner, ermöglicht also Transparenz und Kontrolle in Bezug auf Zugriff, Transfer und Verwendung der eigenen Daten.

**Figure 5:**
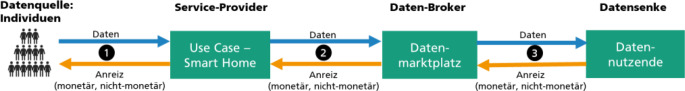

Individuelle Souveränität über persönliche Daten beschränkt sich bisher häufig darauf, die Daten nicht zu teilen und Zugriff zu verwehren. Im Sinne des eingangs formulierten Ausgleichs individueller und gesellschaftlicher Interessen berücksichtigt die Digital Life Journey auch das ökonomische Potenzial der Daten durch ihre Erzeugung, Sammlung, Haltung, Verarbeitung, Verteilung, Analyse, Aufbereitung, Lieferung und Verwertung (siehe Abb. 5).

Das häusliche Umfeld ist ein wichtiger Anwendungsbereich der Digital Life Journey. Immer mehr Unternehmen bieten intelligente und vernetze Komponenten, um das häusliche Lebensumfeld mit Sensorik und Verfahren des maschinellen Lernens auszustatten. Das „Smarthome“-Ökosystem umfasst eine Vielzahl heterogener Systemanbieter von IoT-Hardware und Sensorik über smarte Geräte wie Amazon Echo bis hin zu umfangreichen Systemlösungen. Die Bewohnenden eines Hauses bzw. einer Wohnung und die darin installierten Systeme bilden eine Datenquelle von hohem ökonomischem Potenzial. Voraussetzung ist die Interoperabilität der Daten zwischen Systemen verschiedener Anbieter. Grundsätzliche Nutzenpotenziale sind:

- Individualisierung der einzelnen Dienste durch eine herstellerübergreifende Datennutzung von z. B. Energiedaten, Raumnutzung, Geräteinformationen und anonymisierten personenbezogenen Daten wie Alter, Geschlecht, Wohnort.
- Ermöglichung von Zusatzdiensten durch Pflegedienste, Energieversorger, Stadtplaner, Gesundheitsversorger und Feuerwehr.

In beiden Fällen profitieren – im Sinne des Ökosystembegriffs – sowohl die einzelnen Nutzenden als auch die Gesamtheit der Dienstanbietenden. Im Sinne der Datensouveränität der Datengebenden bzw. der Dienstnutzenden muss das häusliche Umfeld weiterhin als privater Rückzugsraum wahrgenommen werden und der Mehrwert für das Individuum muss ersichtlich sein. Das Individuum muss die Steuerung der Datennutzung durch Dritte sowie die Freiheit Berechtigungen zur Datennutzung selbstbestimmt zu verwalten, d. h. zu gewähren aber auch wieder zu entziehen, behalten. Dies ist der anvisierte Gestaltungsfreiraum, gleichzeitig aber auch -risiko derartiger Initiativen: Treibende erhoffen sich natürlicherweise auch wirtschaftliche Vorteile, wobei sichergestellt werden muss, dass die Interessen der Individuen und Datengebenden ausreichend vertreten und berücksichtigt werden.

## Handlungs- und Forschungsbedarfe

Den aktuellen Entwicklungen und Diskussionen in Europa zur Entwicklung von gemeinschaftlichen Dateninfrastrukturen und Datenräumen liegt die Prämisse zugrunde, eine ausgewogene Balance zwischen Gemeinwohl- und Einzelinteressen im Umgang mit und in der Nutzung von Daten zu finden. Zudem ist es das Ziel, ökonomische Machtkonzentration zu verhindern, wie sie typischerweise in der Plattformökonomie zu beobachten sind. Ein aktuelles Beispiel ist der sogenannte „Data Governance Act“ der Europäischen Kommission, der Regelungen für Datentreuhänder und andere Intermediäre beim Teilen von Daten trifft [8].

Grundlegende Entwurfsprinzipien sind daher neben Datensouveränität, Interoperabilität und Portabilität auch die Verteiltheit der Systeme – und zwar sowohl in Bezug auf die informationstechnische Plattform selbst als auch in Bezug auf Trägerorganisation, Steuerung und Governance. Im Gegensatz zur Entwicklung klassischer Plattformen, die im Wesentlichen durch eine einzelne „Keystone“-Unternehmung finanziert, entwickelt und bereitgestellt werden, bilden sich in Europa viele konsortiale Initiativen heraus. Auch die IDS-Initiative ist ein Beispiel einer allianzbasierten Plattform [19].

Bei der Umsetzung von Datenräumen und Dateninfrastrukturen ergeben sich daraus offene Fragen, sowohl bei europaweiten Initiativen wie GAIA‑X als auch bei nationalen Vorhaben wie dem Datenraum Mobilität sowie einzelnen Projekten wie der Digital Life Journey. Grob lassen sich ethische, rechtliche, betriebswirtschaftliche und technische Fragen unterscheiden.

Beispiele ethischer Fragen sind:

- Wie können die Interessen von Individuen und Gruppen im Sinne des Gemeinwohls im Aufbau von Datenräumen berücksichtigt und vertreten werden?
- Wer hält das Eigentum an Daten: Daten erzeugende Dienstleister, verarbeitende und damit wertschöpfende Unternehmen, oder das Individuum selbst? Wie kann das Individuum dann an der Wertschöpfung entlang der eigenen Daten beteiligt werden, und was sind die eigenen Daten wert?
- Wie können Gesellschaften und Individuen geeignete Digital- und Datenkompetenz aufbauen, um Informiertheit und Souveränität bei der Entscheidungsfindung in Bezug auf die eigenen Daten zu gewährleisten?

Beispiele rechtlicher Fragen sind:

- Was ist die geeignete Rechtsform für Trägergesellschaften von Datenräumen und Dateninfrastrukturen? Gemeinnützige Gesellschaftsformen ohne Gewinnerzielungsabsicht? Oder Genossenschaften in Form beispielsweise der „Societas Cooperativa Europaea“?
- Welche Geschäftsmodelle beim Teilen von Daten unterliegen im Einzelnen europäischer Gesetzgebung und Vorgaben, wie z. B. dem Data Governance Act? Welche Implikationen ergeben sich daraus für die verschiedenen Teilnehmenden an Datenräumen, die ggf. nicht nur Datengebende oder Datennutzende sind, sondern zugleich auch Dienstanbietende?
- Welche Governance-Modelle erlauben einerseits die Abbildung verschiedener Interessen in der Gesellschafterstruktur und schmälern nicht andererseits die Steuerbarkeit der Organisation?

Beispiele für betriebswirtschaftliche Fragen sind:

- Wie finanziert sich der Datenraum- und Dateninfrastrukturbetrieb sowohl während der Aufbau- als auch während der Nutzungsphase?
- Wie sind unverhältnismäßige Belastungen für erste Nutzergruppen zu vermeiden, damit sie nicht überproportional am Aufwand für die Errichtung des Datenraums bzw. der Dateninfrastruktur beteiligt werden? Soll der Staat wie bei anderen Infrastrukturen Teile der Finanzierung übernehmen und, falls ja, in welchem Umfang?
- Was sind wichtige Geschäftsmodellelemente konsortialer Datenraumunternehmen? Gibt es Geschäftsmodellmuster?
- Was sind geeignete Anreizsysteme, um schnell eine kritische Masse an Teilnehmenden für Datenräume zu erreichen?
- Wie sind die Eintrittsbarrieren für Nutzende (Datengebende und Datenempfangende) von Datenräumen niedrigzuhalten und gleichzeitig das Wertversprechen von Datenräumen (Vertrauensschutz, Interoperabilität, Datensouveränität) aufrechtzuerhalten?

Bespiele für technische Fragen sind:

- Wie kann Data Governance in Datenökosystemen umgesetzt werden, wenn Instrumente der „Hierarchie“ (z. B. Arbeitsanweisungen, API-Vorgaben, Prozessdefinitionen), wie sie aus unternehmensinternen Anwendungen bekannt sind, nicht zur Verfügung stehen?
- Wie sind Vertrauensketten in offenen, dynamischen Datenökosystemen zu implementieren?
- Wie gelingt es, Technologien und Konzepte zur Datennutzungskontrolle (z. B. Distributed Usage Control, Open Policy Agent) als Standard in moderne Cloud-Architekturen zu integrieren?
- Welche Rolle spielen Open-Source-Software-Implementierungen?

Die Beantwortung dieser Fragen erfolgt derzeit parallel zur Umsetzung von Datenräumen und Dateninfrastrukturen und kann nur gemeinsam und unter Einbindung sämtlicher Interessensgruppen aus Politik, Wirtschaft und Gesellschaft erfolgen. Dieser europäische Ansatz unterscheidet sich dadurch auch von dirigistischen Strategien einerseits und monopolistisch wirkenden Entwicklungen andererseits, wie sie in anderen Wirtschaftsräumen außerhalb des europäischen Binnenmarkts zu beobachten sind.

## Footnotes

1 Siehe https://www.internationaldataspaces.org/.

2 Open-Source-Software-Implementierungen des IDS-RAM finden sich unter: https://github.com/International-Data-Spaces-Association.

3 Einzelheiten zum IDS Connector sind in DIN SPEC 27070 beschrieben [4].

4 Siehe https://www.data-infrastructure.eu/GAIAX/Navigation/EN/Home/home.html.

5 Siehe https://solidproject.org/.

6 Siehe https://www.digitallifejourney.de/.

## References

[1] Abiteboul S, Agrawal R, Bernstein P (2005). The Lowell database research self-assessment. Commun ACM.

[2] Attoresi M, Moraes T (2020). Personal information management systems.

[3] Die Bundesregierung (2021). Datenstrategie der Bundesregierung.

[4] DIN Standardisiertes Security Gateway und Security-Anforderungen für IoT-Geräte im Small Business/Home-Umfeld (DIN SPEC 27070). Beuth

[5] Eitel A, Jung C, Brandstädter R (2021). Usage Control in the International Data Spaces.

[6] Estermann B, Fraefel M, Neuroni AC (2018). Conceptualizing a national data infrastructure for Switzerland. Inf Policy.

[7] Europäische Kommission (2020). Eine europäische Datenstrategie.

[8] European Commission (2020). Regulation of the European parliament and of the Counctil on European data governance.

[9] Franklin M, Halevy A, Maier D (2005). From databases to dataspaces. Sigmod Rec.

[10] Gelhaar J, Groß T, Otto B (2021). A taxonomy for data ecosystems. Hawaii international conference on system sciences 2021.

[11] Halevy A, Franklin M, Maier D, Gottlob G, van den Bussche J (2006). Principles of dataspace systems. Proceedings of the twenty-fifth ACM SIGMOD-SIGACT-SIGART symposium on Principles of database systems—PODS ’06.

[12] Hummel P, Braun M, Tretter M (2021). Data sovereignty: a review. Big Data Soc.

[13] IDS Association, Fraunhofer (2021). GAIA-X and IDS.

[14] Kokolakis S (2017). Privacy attitudes and privacy behaviour: a review of current research on the privacy paradox phenomenon. Comput Secur.

[15] Lansdale MW (1988). The psychology of personal information management. Appl Ergon.

[16] Mansour E, Sambra AV, Hawke S, Bourdeau J, Hendler JA, Nkambou RN (2016). A demonstration of the solid platform for social web applications. WWW’16 companion.

[17] Meister S, Otto B (2019). Digital life journey.

[18] Nahles A (2019). Digitaler Fortschritt durch ein Daten-für-Alle-Gesetz.

[19] Otto B, Jarke M (2019). Designing a multi-sided data platform: findings from the International Data Spaces case. Electron Markets.

[20] Otto B, Lohmann S, Steinbuß S (2019). IDS reference architecture model (version 3.0).

[21] Otto B, ten Hompel M, Wrobel S, Neugebauer R (2019). International data spaces. Digital transformation.

[22] Pistor K (2020). Rule by data: the end of markets?. Law Contemp Probl.

[23] Pullmann J, Petersen N, Mader C (2017). Ontology-based information modelling in the industrial data space. 22nd IEEE International Conference on Emerging Technologies and Factory Automation (ETFA).

[24] Teevan J, Jones W, Czerwinski M (2008). The disappearing desktop. CHI ’08 extended abstracts on human factors in computing systems.

[25] W3C Data Catalog Vocabulary (DCAT)—Version 2. https://www.w3.org/TR/2020/REC-vocab-dcat-2-20200204/. Zugegriffen: 30.7.2021

